# Power Intelligent Terminal Intrusion Detection Based on Deep Learning and Cloud Computing

**DOI:** 10.1155/2022/1415713

**Published:** 2022-05-09

**Authors:** Tong Li, Hai Zhao, Yaodong Tao, Donghua Huang, Chao Yang, Shuheng Xu

**Affiliations:** ^1^College of Computer Science and Engineering, Northeastern University, Shenyang 110169, China; ^2^Liaoning Electric Power Research Institute of State Grid Corporation of China, Liaoning 110055, China; ^3^Beijing DualPi Intelligent Security Technology Co. Ltd., Beijing 100088, China; ^4^State Grid Liaoning Electric Power Co., Ltd., Liaoning 110004, China

## Abstract

Numerous internal and external intrusion attacks have appeared one after another, which has become a major problem affecting the normal operation of the power system. The power system is the infrastructure of the national economy, ensuring that the information security of its network not only is an aspect of computer information security but also must consider high-standard security requirements. This paper analyzes the intrusion threat brought by the power information network and conducts in-depth research and investigation combined with the intrusion detection technology of the power information network. It analyzes the structure of the power knowledge network and cloud computing through deep learning-based methods and provides a network interference detection model. The model combines the methods of abuse detection and anomaly detection, which solves the problem that the abuse analysis model does not detect new attack variants. At the same time, for big data network data retrieval, it retrieves and analyzes data flow quickly and accurately with the help of deep learning of data components. It uses a fuzzy integral method to optimize the accuracy of power information network intrusion prediction, and the accuracy reaches 98.11%, with an increase of 0.6%.

## 1. Introduction

Like most data technologies, cloud computing has become a hot topic in the global computer electronics industry. Cloud computing is closely integrated with computer technology and network. This is another development of data technology other than the Internet and computers. It will provide users with powerful computing power and sufficient storage space. Cloud end users only need mobile devices to manage their various cloud data resources, while cloud computing is open [1, 2]. The cloud computing method is more complicated than the traditional computing network, the time is long, and the management is more difficult. Hackers only need smart mobile devices to create a crowded network environment for cloud computing and cause massive damage. There are also cybersecurity concerns from within, for example, in the daily office platform of the power grid integrated information network and the external power transaction business and application platform. Threats such as illegal access within the network, abuse of network resources, wanton spread of viruses, and application and system vulnerabilities have seriously affected the normal business and application operations of the power information system. Therefore, the behavior problem of smart mobile devices in cloud computing has become a network security problem. And because the way of intelligent mobile terminal intrusion detection in cloud computing has had an irreversible impact on the security of cloud computing environment, the necessity of research is very high, which has become a major topic of current research.

The main intrusion detection methods of mobile intelligent terminals in today's cloud computing include intrusion detection methods based on neural network algorithms, intrusion detection methods based on vector-assisted algorithms, and intrusion detection methods based on undefined algorithms. The most common is invasive detection based on uncertain clustering algorithms. Periodic algorithms are suitable for most intrusion detection, but they also have shortcomings. Vector network algorithms and vector support require a large amount of disturbing sample data for intrusion detection training. But the interference environment data model is still a small sample set, so it will reduce the accuracy of intrusion detection. The intrusion detection method based on fuzzy clustering algorithm is very sensitive to the choice of initial value.

Aiming at the shortcomings of the above traditional algorithms, this paper proposes a mobile terminal intrusion detection method in the cloud computing environment. It calculates the similarity of interference data in the cloud computing environment, calculates the posterior probability of each disturbance data attribute feature in the Bayesian model, updates the calculation result to the initial probability, and processes the interference data in the process of intrusion detection. It reduces the correct classification of interference data to the highest probability obtained in order to achieve accurate interference data detection results. The simulation results show the superiority of the improved algorithm.

## 2. Related Work

Experts at home and abroad also have many research results in the intrusion detection of power intelligent terminals in deep learning and cloud computing [3, 4]. Xia et al. extensively studied content-based image retrieval (CBIR). Images consume more storage space than text documents. Therefore, its maintenance is considered as a typical example of cloud storage outsourcing [5]. Deng et al. extended cloud computing by deploying localized computing facilities in advance at users, prestoring, and distributing cloud data to mobile users with fast local connections. Therefore, fuzzy computing introduces an intermediate layer between mobile users and the cloud to complement cloud computing to provide low-latency, high-rate services to mobile users [6]. Wei et al. believe that the existing static grid resource scheduling algorithms are limited to minimizing the completion time and cannot meet the requirements of cloud computing for resource scheduling. Current cloud infrastructure solutions only provide operational support at the resource infrastructure level. When hardware resources form a virtual resource pool, virtual machines are deployed and used transparently [7]. Hirai et al. believe that, in cloud computing, large-scale parallel distributed processing services are provided. One of the giant tasks is split into multiple subtasks, which are processed on clusters of machines called workers. In such processing services, it takes a long time to process subtasks, resulting in long response times [8]. Chen et al. believe that stacked autoencoders, as a deep learning architecture, aim to obtain useful high-level features. Extensive experimental results using hyperspectral data show that a deep learning-based framework is constructed to provide comparisons for classifiers [9]. Kermany et al. built a diagnostic tool based on a deep learning framework that utilizes transfer learning to train neural networks with a fraction of the data from traditional methods [10]. However, due to the lack of relevant cloud computing data in these studies, there are also some controversies in the methods used, resulting in the relevant results not being recognized by the public.

## 3. Theories and Key Technologies of Intelligent Terminal Intrusion Detection

### 3.1. XManDroid Model

**Figure 1:**
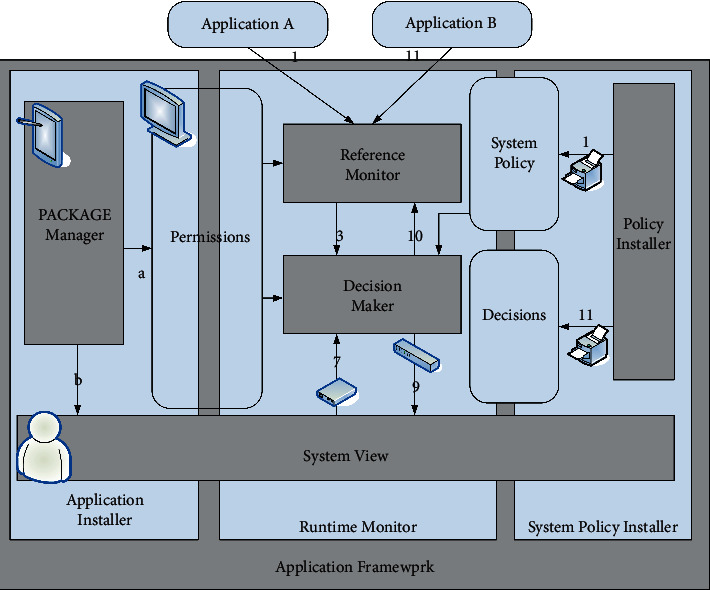
XManDroid architecture diagram.

XManDroid is a security model based on a system detection strategy for running monitoring and preventing application layer privilege escalation. It makes an allow or deny decision by examining the data transfer and intent content in the ICC. Among them, there are mainly installation strategies, execution strategies, matching strategies, search strategies, and program uninstall strategies that need to be formulated to implement. The architecture of XManDroid is shown in Figure 1 [11].

### 3.2. Hybrid Intrusion Detection Model Based on DBN and TSVM

The intrusion detection system makes an appropriate response according to the result of abnormal processing and judgment of the data, such as terminating the process or just issuing an alarm. The working mechanism of the response module is generally divided into the following two types:

1. Active response: when an intrusion behavior is identified, the system can take the initiative to take some defensive actions to deal with the attack behavior, such as terminating the process [12, 13].
2. Passive response: this kind of response simply records the intrusion behavior, the system does not take the initiative to do specific defense operations, and the defense operations are decided by the network administrator. When the intrusion detection model designed in this paper detects an intrusion, it first issues an alarm to the network administrator. If the detection is a known intrusion type, the identification result is given. If unknown, give the approximate range for the reference of network administrators to take further measures.

**Figure 2:**
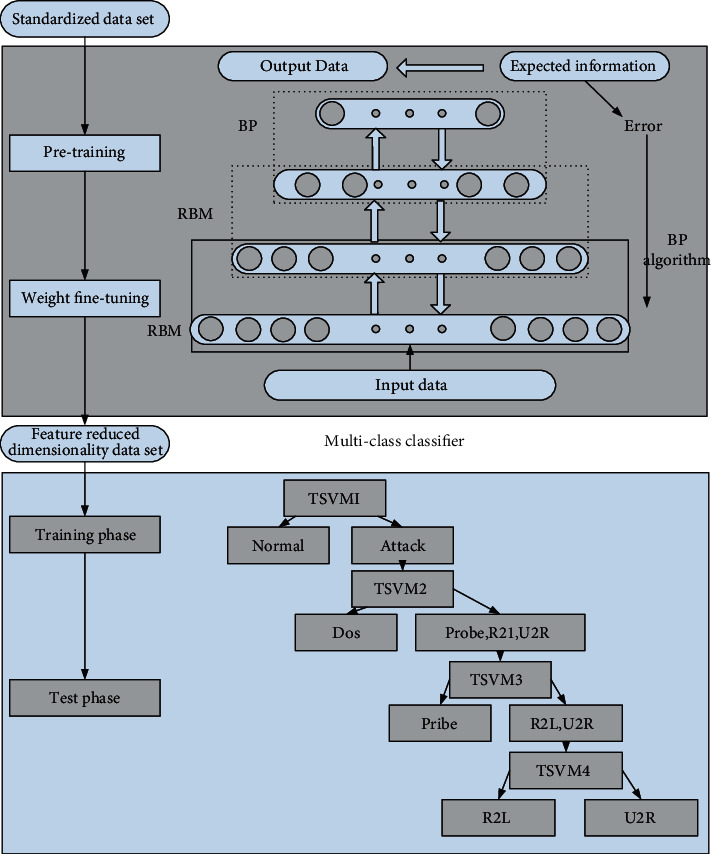
Exception handling module design based on DBN and TSVM.

Figure 2 shows the design of the exception handling module based on DBN and TSVM.

### 3.3. Host-Based and Network-Based IDS

**Figure 3:**
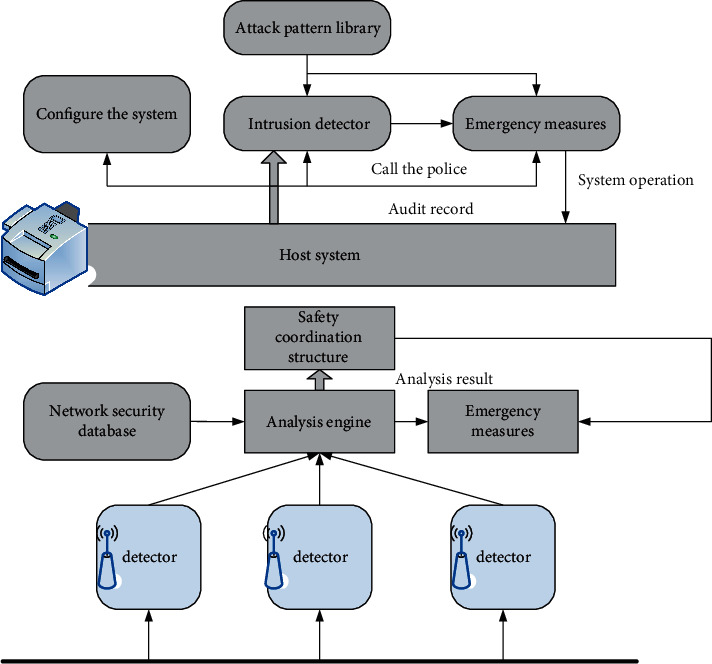
Network-based intrusion detection system structure.

According to the different sources of data used by IDS, it can be divided into Host-based IDS and HIDS. HIDS generally builds detection modules on the protected host system. These detection modules are usually some client programs, which can extract audit records or log files on the protected computer system and analyze them according to certain rules, as shown in Figure 3. The key point of IDS is to audit and analyze the data provided by the host system and find the evidence of abnormal system activity, so as to identify the intrusion behavior and make the appropriate response in time. Because such IDS systems will be limited by the host, targeted and vulnerable to attack, HIDS is generally specially formulated for a specific system, and its compatibility is poor [14].

### 3.4. Intrusion Detection Model of Power Management Information Area Network

**Figure 4:**
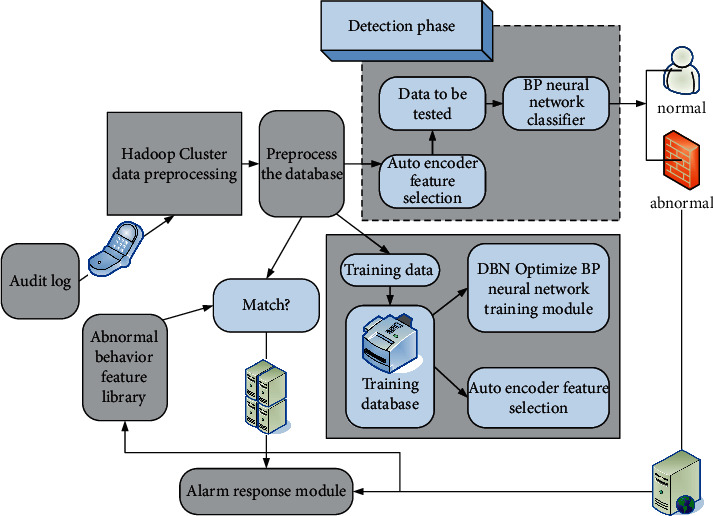
Power information network intrusion detection model.

The intrusion detection cloud center is divided into two stages: real-time induction and detection training. If the training step is set, the collected data has been processed by the Hadoop cloud platform. It takes the preprocessed data as the input data and trains the multilayer encoder to capture the data features, and the initial pressure is to optimize the BP network at the same time. In the actual detection step, the behavior record part is passed to the BP neural network classifier, and the alarm information is generated according to the result of the BP classifier. The figure shows the power information network intrusion detection model based on the deep learning algorithm under the cloud platform.  Figure 4 shows the intrusion detection model of the power information network.

### 3.5. The Basis of Mobile Intelligent Terminal Intrusion Detection

Through the experimental analysis of malicious samples, the permission list and attack behavior description of several typical malware applications are shown in Table 1.

Analysis of malware samples: the more the permissions are requested, the more space the malware program loses and the greater the threat to users is. Before the attack, many malware had already obtained multiple system permissions and successfully requested API threats.

**Figure 5:**
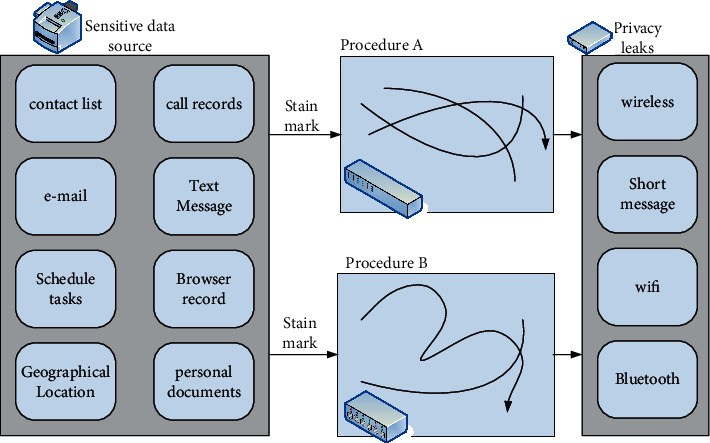
Schematic diagram of dynamic tracking technology.

Dynamic taint analysis is a technology that extracts data from mobile smart terminals. Scholars formally put forward the idea of “taint propagation analysis” in their research to solve the gap problem. The dynamic analysis of tainted multiplication is to look for programs that normally run and may have unsafe conditions if they receive input from external data. The identification process includes three processes: marking tainted data, tracking tainted data, and identifying tainted data. Among them, tainted data is the reason why programs receiving external data may be attacked, but the data is not necessarily malicious. With the rise of mobile smart terminals, there are more and more privacy leakage behaviors in the application software in smart terminals. In order to obtain tainted data that leads to privacy leakage, tainted data analysis technology is widely used in the privacy leakage of smart terminals [15, 16]. Taking the smartphone with Android operating system as an example, the principle of dynamic tracking technology is shown in Figure 5 [17].

### 3.6. Restricted Boltzmann Machines

**Figure 6:**
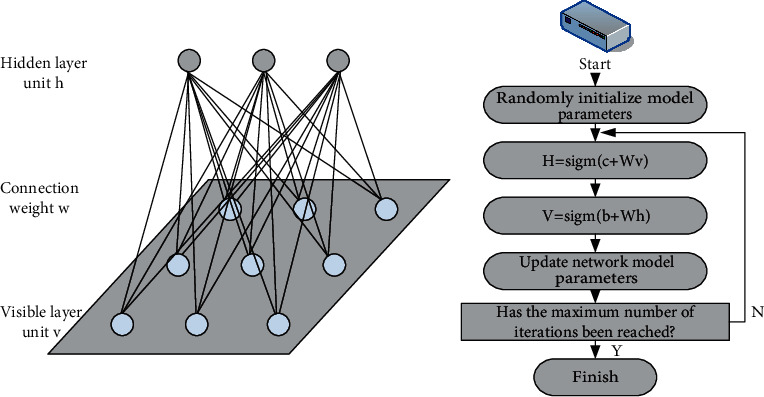
The structure diagram of the RBM network and the flow chart of the CD-based fast learning algorithm.

Restricted Boltzmann Machine (RBM) was proposed in 1986, which is a new type of stochastic neural network model. Compared with the traditional Boltzmann machine, the network structure of RBM is a bipolar graph, which has no side links in the visible layer and no hidden links in the hidden layer. There are only edge connections between visible layer units and hidden layer units, as shown in Figure 6 [18].

As can be seen from Figure 6, in the RBM network model diagram, assuming that the number of nodes in the visible unit (*v*) is greater than the number of characters in the hidden unit (*h*) in *m*, each visible node *v* has only *m* hidden nodes. Unlike other visualizations, there are no relationships between nodes. So, each hidden node affects only *n* visible nodes, and the value range of *v* and *h* is {0, 1} [19].

The energy function *E* (*v*, *h*) of RBM is

(1) $$\begin{array}{c} E\left(v,h\right)=-\underset{ij}{\sum }W_{ij}v_{i}h_{j}-\underset{i}{\sum }b_{i}v_{i}-\underset{j}{\sum }c_{j}h_{j}. \end{array}$$

Among them, *v*_*i*_ is the visible layer unit, *h*_*j*_ is the hidden layer unit, *b*_*i*_ is used to represent the deviation of *v*_*i*_, *c*_*j*_ is used to represent the deviation of *h*_*j*_, and *W*_*ij*_ represents the weight between *v*_*i*_ and *h*_*j*_.

In the RBM network, we can calculate the joint probability distribution according to the Gibbs distribution:

(2) $$\begin{array}{c} p\left(h|v\right)=\prod_{i=1}^{m}p\left(h_{i}|v\right), \end{array}$$

(3) $$\begin{array}{c} p\left(h|v\right)=\prod_{i=1}^{m}p\left(v_{i}|h\right). \end{array}$$

Because there is no connection between hidden layer units, the marginal distribution calculation of the above formula is very simple:

(4) $$\begin{array}{c} p\left(v\right)=\frac{1}{z}p\left(v,h\right)=\frac{1}{z}\prod_{i=1}^{n}e^{h_{i}v_{i}}\prod_{i=1}^{m}\left(1+e^{c_{j}+\sum_{i=1}^{n}w_{ij}v_{i}}\right)p\left(h_{i}|v\right). \end{array}$$

Similarly, (ph) can also be calculated.

According to Bayes' principle, knowing the joint probability and the marginal probability, the conditional probability can be calculated:

(5) $$\begin{array}{c} p\left(h_{j}=1|v\right)=sigm\left(\overset{n}{\underset{i=1}{\sum }}w_{ij}v_{i}+c_{j}\right). \end{array}$$

(6) $$\begin{array}{c} p\left(v_{j}=1|v\right)=sigm\left(\overset{n}{\underset{j=1}{\sum }}w_{ij}h_{j}+b_{i}\right). \end{array}$$

Given training sample *S*={*v*_1_, *v*_2_, ..., *v*_*N*_}, since the training data are independent of each other, the goal of training RBM is to maximize the following likelihood function:

(7) $$\begin{array}{c} L\left(\theta \right)=\overset{N}{\underset{n=1}{\sum }}1np\left(v_{n};\theta \right), \end{array}$$

(8) $$\begin{array}{c} \overset{N}{\underset{n=1}{\sum }}1n\underset{h}{\sum }p\left(v_{n},h;\theta \right), \end{array}$$

(9) $$\begin{array}{c} \overset{N}{\underset{n=1}{\sum }}\left(1n\underset{h}{\sum }\exp\left\{-E\left(v_{n},h;\theta \right)\right\}-1n\underset{v,h}{\sum }\exp\left\{-E\left(v,h;\theta \right)\right\}\right), \end{array}$$

where *θ*={*W*, *b*, *c*} is the RBM network model parameter. Generally, a stochastic gradient is performed on the log-likelihood of the input sample, and the optimal RBM network model can be obtained.

(10) $$\begin{array}{c} \frac{\partial L\left(\theta \right)}{\partial \theta }=\frac{\partial }{\partial \theta }\overset{N}{\underset{n=1}{\sum }}\left(1n\underset{h}{\sum }\exp\left\{-E\left(v_{n},h;\theta \right)\right\}-1n\underset{h}{\sum }\exp\left\{-E\left(v_{n},h;\theta \right)\right\}\right). \end{array}$$

(11) $$\begin{array}{c} \overset{N}{\underset{n=1}{\sum }}\left(\frac{\partial }{\partial \theta }1n\underset{h}{\sum }\exp\left\{-E\left(v_{n},h;\theta \right)\right\}-\frac{\partial }{\partial \theta }1n\underset{v,h}{\sum }\exp\left\{-E\left(v_{n},h;\theta \right)\right\}\right), \end{array}$$

(12) $$\begin{array}{c} -{\overset{N}{\underset{n=1}{\sum }}\langle \frac{\partial E\left(v_{n},h;\theta \right)}{\partial \theta }\rangle }_{p\left(h|v_{n}\right)}+\overset{N}{\underset{n=1}{\sum }}{\langle \frac{\partial E\left(v_{n},h;\theta \right)}{\partial \theta }\rangle }_{p\left(h|v\right)}, \end{array}$$

where 〈...〉 in the above formula represents the expected value in probability theory. From the conditional independence of RBM, we can analyze that the first term in formula (12) can be solved. The second term is unsolvable due to the existence of *z*(*θ*) and is generally represented by sampling methods.

If there is only one training data sample, *p*(*hv*_*n*_) and *p*(*v|h*) are simplified as “data” and “model.” Then, the partial derivative of the log-likelihood function for each component of parameter *L*(*θ*) can be expressed by formula

(13) $$\begin{array}{c} \frac{\partial L\left(\theta \right)}{\partial w_{ij}}={\langle v_{i}h_{j}\rangle }_{\text{data}}-{\langle v_{i}h_{j}\rangle }_{\text{model}}. \end{array}$$

(14) $$\begin{array}{c} \frac{\partial L\left(\theta \right)}{\partial b_{i}}={\langle v_{i}\rangle }_{\text{data}}-{\langle v_{i}\rangle }_{\text{model}}. \end{array}$$

(15) $$\begin{array}{c} \frac{\partial L\left(\theta \right)}{\partial c_{i}}={\langle h_{j}\rangle }_{\text{data}}-{\langle h_{j}\rangle }_{\text{model}}. \end{array}$$

In this paper, a feature learning algorithm based on deep belief network is designed, and the influence of the structural model of DBN on its feature learning ability is analyzed. The experiment will compare the feature learning ability of DBN with the classical feature learning methods, such as principal component analysis PCA, information gain, gain ratio, and other methods. In the experiment, the data sets S1, S2, S3, and S4 selected from KDDCUP'99–10% are also used as the original data sets. The above-mentioned feature representation method is used to extract features from the dataset, and then SVM is used to classify the new feature data [20–23].

The data in Table 2 are referenced from Wang et al. (Network Intrusion Detection Based on Deep Learning) [24]. Through the experimental comparison results given in Table 2, it is found that the DBN-based feature learning method performs intrusion detection training on four different data sets, and all beat the traditional feature learning method by a large advantage. For example, for the large-scale dataset S4, the feature learning method based on DBN is 11.58% higher than the PCA method and 12.91% higher than the gain ratio method. Therefore, feature learning algorithms based on DBN are more suitable for feature learning tasks in high-dimensional spaces.

Firstly, the traditional feature learning method is studied, and its inefficiency in the face of massive data is analyzed. Then the BP network and RBM network algorithms are introduced in detail. Finally, a new feature learning algorithm based on DBN is proposed, and the influence of the parameters of the DBN network model on the intrusion detection effect is also analyzed through experiments. The experimental comparison between the DBN-based deep feature learning model and other traditional common feature learning methods is carried out. In the experimental part, some basic information of the KDDCUP'99 dataset is briefly summarized, including its source, data type, data format, and the meaning of feature attributes. Then, the method of preprocessing the original data set is introduced in detail, including numerical processing of character data samples to facilitate machine identification and normalization of data to facilitate subsequent classification and detection [25, 26].

## 4. Test Data for Intelligent Terminal Intrusion Detection

The data set used in this paper is the network traffic audit log data of a power company. In order to increase the data of various intrusion behaviors, a part of the security audit data set is added to the data set analyzed and processed by Hadoop to form the intrusion detection data set (KD). The data set has a total of 2 million network traffic as training data, and the test data set has a total of 1 million data sets. It contains four types of intrusion: Dos attack, port scan attack, unauthorized access by remote host, and unauthorized access by local user. There are 39 types of intrusion attacks in total, 22 types of intrusion types are included in the training data set, and each record has a total of 53-dimensional attributes; the last attribute is the category. The data are combined from several aspects. First, consider the basic characteristics of network connections, such as source IP address, destination IP address, source port, destination port, and other attribute fields. Second, consider the content characteristics of the network connection: the data part of the data packet contains information such as the user's remote access and operating system sensitive files instructions and the password for logging into the system. Third, consider the temporal characteristics of traffic: due to the time correlation of network attacks, some connections with the connection within 2 s before the current connection are counted, such as the percentage of the same host and service type as the current connection in the first 2 s. Fourth, consider the traffic statistics characteristics for a specific host: the actual network attack behavior will be longer than the 2 s time span. In order to find out this part of the attack, the relationship between the 100 connections before the current connection and the connection is counted, for example, the percentage of the first 100 connections that have the same host and service type as the current connection [27].

Because the data contains two types of discrete and continuous data, and in order to fit the input of neurons and eliminate the situation that large numbers eat small numbers, it is necessary to standardize and normalize the data.

(1) The input of the data normalization neural network requires numerical input, and the items whose attribute fields are character types are uniformly encoded. The encoding method adopts the lexicographical sorting method to assign ordinal numbers to the character fields in turn. If there are three types of protocols: TCP, UDP, and ICMP, the results sorted according to the lexicographical order of the fields are ICMP, TCP, and UDP. The codes are shown in Table 3; other character fields are normalized in the same way [28]. Table 3 is reproduced from Wang et al. (under the Creative Commons Attribution License/public domain).
(2) The size range of the data normalization attribute is reduced to the [0, 1] interval, and the paper uses formula (16) to normalize the data.
  (16) $$\begin{array}{c} a=\frac{a-\min}{\max-\min}, \end{array}$$
where a represents the value of the attribute field and max and min represent the maximum and minimum values of the attribute field.

### 4.1. Description of Evaluation Indicators

(1) Suppose there are a total of *m* normal network behaviors and *n* attack behavior data sets of test samples. After the trained intrusion detection model, m' normal network behavior records and *n*' attack behavior records are correctly identified. Then the overall correct rate (Rc), false-positive rate (Rw), and false-negative rate (Rl) of the test sample data set are
  (17) $$\begin{array}{c} R_{c}=\frac{m^{\prime }+n^{\prime }}{m+n}, \end{array}$$
  (18) $$\begin{array}{c} R_{w}=\frac{m-m^{\prime }}{m}, \end{array}$$
  (19) $$\begin{array}{c} R_{l}=\frac{n-n^{\prime }}{n}. \end{array}$$
(2) The parallel performance evaluation of the algorithm adopts the speedup index and the scaleup index. Speedup ratio is an index to measure the performance and effect of parallel system or program parallelization; the formula is shown in
  (20) $$\begin{array}{c} \text{speedup}=\frac{T_{\text{Stand}-\text{alone}}}{{\text{T}}_{\text{Cloudcluster}}}. \end{array}$$

In the formula, *T*_stand alone_ is the time spent by the task to run on a single-processing system. T_clound cluster_ is the time spent by tasks running on the cluster. The scaling rate is the ratio of the running time of executing a task on a cluster with m-fold more nodes and an m-fold increase in data volume to the running time of the algorithm running the original dataset.

(21) $$\begin{array}{c} \text{scaleup}=\frac{T_{\text{dataset}}}{T_{\text{Originaldataset}}}. \end{array}$$

In the formula, *T*_data set_ is the running time of the algorithm after the data set is expanded by *m* times; *T*_original data set_ is the running time of the algorithm on the original data set; *m* is the multiplier of the data set enlargement.

### 4.2. MR_SAE Algorithm Experiment

A total of 202,860 Dos attacks and normal unlabeled data in the KD dataset are selected for the training of the autoencoder. Then test with 102,503 data sets containing 17 unknown intrusion types, and determine the number of hidden layer nodes of the autoencoder by a bisection method.  Table 4 shows the correspondence between the number of hidden layer nodes and the mean square error and running time of the autoencoder after 500 iterations.

As the number of hidden nodes increases, the error gradually increases, and when the number of hidden layer nodes is 20 to 25, the error begins to decrease again. Since the number of hidden nodes is [5, 10], the error is the smallest, and the running time is the shortest. Therefore, the interval of the number of nodes is set to [5, 9], traversing one by one, and the experimental results are shown in Table 5.

After the training of the autoencoder is completed, the detection rate of the BP neural network with a different number of hidden layer nodes of the encoder is combined with the BP neural network experiment.

The detection rate of this method compared with other methods is shown in Table 6. It can be seen that the autoencoder algorithm is superior to the other three algorithms in detection rate and can utilize a large amount of unlabeled data on the network.

**Figure 7:**
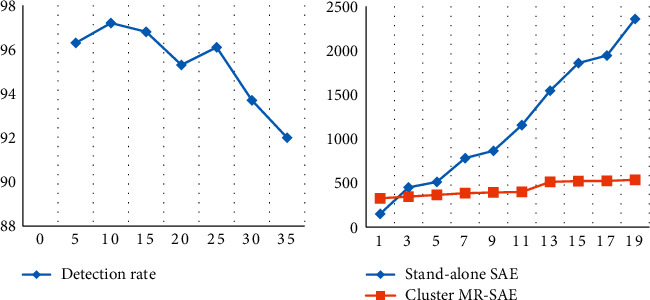
Different hidden node detection rates of autoencoder HE single-machine SAE and 4-node cloud cluster MR_SAE.

The extraction performance of the MR_SAE algorithm is tested under different data volumes. The KD data set data were randomly generated into multiple sets of data sets, namely, KD1, KD2, KD3, KD4, KD5, KD6, KD7, and KD8. Among them, KD1 has 10,000 pieces of sample data, KD2 has 30,000 pieces of data, KD3 has 50,000 pieces of data, KD4 has 70,000 pieces of sample data, KD5 has 100,000 pieces of sample data, KD6 has 130,000 pieces of sample data, KD7 has 160,000 pieces of sample data, and KD8 has 200,000 pieces of sample data. We compared the training time of the SAE algorithm on a single machine with the training time of MR_SAE in a cluster environment. The comparison results are shown in Figure 7.

With the expansion of the training set size, the time to execute the MR_SAE algorithm on a 4-node cloud computing cluster does not change much. In the stand-alone environment, with the expansion of the training set, the execution time of the SAE algorithm also increases. Through the analysis of the experimental results, it can also be concluded that, in the 4-node cloud computing cluster environment, the training speed of the MR_SAE algorithm is more than three times that of the single-machine SAE algorithm. In practical applications, with the increase of the number of cloud cluster nodes and the growth of the power information network connection record sample data set, the parallel performance of the MR_SAE algorithm based on cluster computing will be better [29].

### 4.3. MR_DBN_BP Algorithm Classification Performance Test

1. Data sample set construction: because there are relatively few R2L and U2R in the entire KD data set, the sample data set is divided into training set and test set according to the ratio of 3 : 1. Among them, 6590 network connection data are randomly selected as the sample data set. The sample data contains continuous and discrete values, so it is necessary to standardize and normalize the data. Encode the category of the record, so that the BP can be classified correctly.
2. The training parameter setting of the experimental method: in order to compare the experimental method with the BP method, PSO-BP, and GA-BP methods that introduce the steepness factor, the network parameters are set as follows:
3. Set the number of RBMs constituting the DBN network to two and the number of nodes to be 53-22-12, and iterate 1000 times.
4. The BP neural network has a three-layer structure, the number of nodes is 12-6-3, the iteration is 500 times, the learning rate is 0.01, and the error rate is 0.013. According to the experimental results and analysis, Table 7 is the comparison with the other three methods in terms of detection rate, false-positive rate, and false-negative rate [31]. Table 7 is reproduced from Wang et al. (under the Creative Commons Attribution License/public domain).

It can be seen from the experiments that the detection time of the BP network optimized based on DBN is slightly longer than that of other methods in the case of a single machine. However, because this method can select more essential features of the data during training, it has higher detection accuracy than other methods and also effectively reduces the false detection rate and missed detection rate [32].

**Figure 8:**
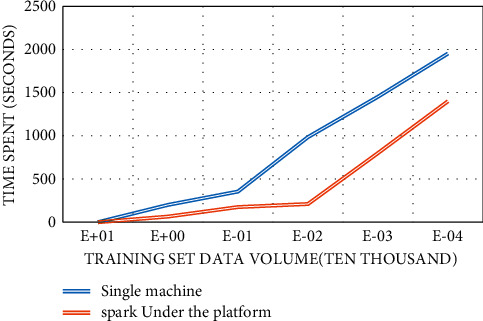
DBN_BP algorithm training comparison relationship.

The relationship between the number of iterations and the mean square error of training the DBN-optimized BP network under a single machine and the comparison of training on Spark are shown in Figure 8.

**Figure 9:**
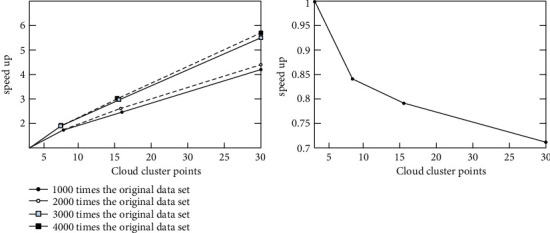
MR_DBN_BP algorithm expansion rate.

As can be seen from Figure 8, it takes longer to train DBN_BP on a single machine, and more iterations are needed to meet the error requirements. On the cluster, the three nodes in the experiment simultaneously train DBN_BP in batches so that fewer iterations can meet the requirements. In order to compare the parallel expansion performance of the cluster, the original 6590 network sample data were manually copied by 1000 times, 2000 times, 3000 times, and 4000 times, respectively. Then, the algorithm proposed in this paper is run on cloud platforms with 4, 8, 16, and 32 cluster nodes, respectively, and finally, the speedup ratio and expansion ratio are calculated. Ideally, the speedup ratio and expansion rate of the cloud computing parallel system are both 1; however, in practical applications, due to the existence of communication delay between nodes, this rational state is impossible to achieve, and with the increase of the data set, the expansion rate of cluster computing will gradually decrease. As the number of cluster nodes increases, the communication overhead between nodes increases gradually, so the speedup ratio of the system also decreases. The experimental results are shown in Figure 9. When the data volume of the experimental data set becomes larger, the acceleration ratio growth rate of the MR_DBN_BP algorithm gradually decreases, and the decreasing slope of the expansion rate of the algorithm becomes smaller. Therefore, the expansion rate index of the MR_DBN_BP algorithm performs better [33].

**Figure 10:**
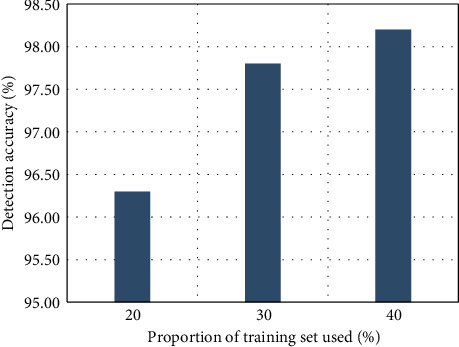
Histogram of DBN-ELM accuracy optimized by the fuzzy integral method.

In order to prove that the multiclassification method based on fuzzy integral fusion is better than the original DBN-ELM and the simple weighting method, on the basis of the trained DBN, three ELMs with different structures are trained, and the fuzzy integral fusion is performed. Then focus on the effect of nontraining at different scales in the NSL-KDD dataset. The final training of 20%, 30%, and 40% NSL-KDD dataset is completed by the fuzzy integral fusion method DBN-ELM and the simple evaluation method and then compared with the test set. The DBN components are designed using the same parameters, as shown in Figure 10. It achieves 98.11% accuracy using DBN-ELM optimized by the integral fuzzy method, which is the result obtained after training 40% of the training set. It can also be seen from the figure that the optimized DBN-ELM maintains its inherent advantages of strong expression ability and good generalization. The results obtained after training from 30% to 40% of the training set are not very different. Due to the advantage of strong generalization ability, only a few data sets are needed to train the ability to identify unknown attacks [34].

The figure shows the DBN-ELM and the posttraining accuracies of the fuzzy integral fusion method and the weighted average method on 20%, 30%, and 40% of the dataset, respectively. It can be seen that the weighted average method is the best in 40% of the data sets compared to the original DBN-ELM, but the improvement effect is limited, and there is a negative impact on 20% of the data sets. However, after optimization using the fuzzy integral method, it has a significant improvement over the original DBN-ELM, and there is no negative impact. It finally achieved 98.11% accuracy, with an improvement of 0.6% accuracy. Because the paper does not train a large number of ELMs with different structures for fusion, it just verifies the effect of the fuzzy integral. Therefore, in practical applications, if a large number of ELMs are fused, it may get a larger result than the original model. It can be seen that, after using fuzzy integral optimization, the overall false alarm rate is also the lowest, while the weighted average method has limited effect and also has a negative impact phenomenon [35].

The Spark cloud computing platform with 4 nodes is built in the laboratory for the experiment and performance test of the two cloud-based deep learning algorithms proposed in this paper. Through the standardization and normalization of the dataset, it is used as the input of the neural network unit. Through the spark -based MR_SAE performance test, this article compares some traditional feature extraction algorithms and BP optimization algorithms. The experimental results show that the classification accuracy and performance after extraction are better than traditional algorithms, and it has good parallelism. The deep learning algorithm based on Spark can meet the huge demand for computer resources when performing quasi-real-time detection on massive high-dimensional power grid intrusion detection data. Although previous approaches using DBN-ELM have yielded fairly good results, a distributor is always biased. Therefore, based on DBN-ELM, the fuzzy integral fusion multiclassification method is used to play the positive role of the distributor, eliminate the negative role of the classifier, further improve the classification ability, and perform intrusion detection on it [36]. Aiming at the problem that the fuzzy dimension is difficult to determine, the idea of dynamic fuzzy measurement is adopted, and the fuzzy dimension is determined by a neural network [37–39]. Finally, experiments were performed on KDD99 and NSL-KDD data. The experimental results show that the multiclassifier method based on fuzzy integral fusion can make DBN-ELM further improve the accuracy of intrusion detection and identification and only increase the training time by a small amount, which proves the effectiveness of the testing method.

## 5. Discussion

Aiming at the shortcomings of traditional mobile intelligent terminal intrusion detection algorithms in cloud computing environment, a mobile intelligent terminal intrusion detection method based on cloud computing environment optimization Bayesian algorithm is proposed. Calculate the similarity of disturbance data in the cloud computing environment, and calculate the posterior probability of each disturbance data attribute feature in the Bayesian model. In the process of intrusion detection, the interference data is processed, and the correct classification of the interference data is reduced to the highest probability of obtaining accurate detection results.

DBN-ELM hybrid model, which is mainly based on large and complex intrusion detection data features, has the ability of unsupervised DBN training and automatic feature retrieval, as well as fast learning features and good ELM generalization ability. First, DBN-ELM uses a large amount of unlabeled intrusion detection data to monitor training at the DBN side, making full use of a large amount of unlabeled intrusion detection data. The trained DBN can map unnecessary semi-low-level features layer by layer into extremely abstract important features. The ELM is trained using a small amount of labeled data for feature extraction based on the trained DBN. ELM algorithm is a fast learning algorithm. The advantage of ELM is that it has good generalization and can detect and identify unknown attacks. The DBN-ELM model combines the capabilities of DBN for unattended and automatic participatory learning with the advantages of fast ELM learning and good generalization. Experiments show that the model can effectively improve the detection accuracy and training speed of intrusion analysis.

In the process of obtaining DBN-ELM, for the problem that the number of network layers is difficult to determine, the depth of the network is determined by changing the correlation between the features of the hidden output layer of DBN-ELM. Because ELM is a hidden layer structure, it is mainly to determine the number of network layers in the DBN part. As the number of network layers increases, the number of correlations between different categories decreases so that when the number of correlations decreases to a certain scale. The number of network layers not only increases the power of the model but also increases the difficulty in determining the number of layers in the network by observing the number of correlated returns.

For intrusion detection methods based on the fusion of multiclassifier fuzzy integrals, there is always a specific tendency to use classifiers for DBN-ELM. It proposes a method to combine multiple classifiers using fuzzy integrals based on DBN-ELM. Allocators of different types and classes of the same type but with different structural parameters always have different deviations. Their dislocation regions are continuous, and there are positive and negative synergistic relationships between them. Therefore, this paper adopts the fuzzy integration method to fuse multiple classifiers to exert the positive synergistic relationship, eliminate the negative synergy relationship, and further improve the accuracy of intrusion detection. The experimental results show that the multiclassifier method based on fuzzy integral fusion further improves the accuracy of intrusion detection and recognition.

Aiming at the problem that fuzzy measurement is difficult to determine, based on the idea of dynamic fuzzy measurement, this paper adopts the neural network method to determine the fuzzy measurement. The traditional fuzzy methods of measurement decision-making use the methods prescribed by experts, which have many limitations or are solved by planning methods, which are more complicated and less effective. And the learning method of using neural network to measure nerve can be dynamic; according to different sample changes, the powerful expressive ability can fully meet the complexity requirements of fuzzy measurement. In the process of fuzzy integration and multiclassifier for intrusion detection, the paper adopts a neural network to learn fuzzy detection. The experimental results show that this method is effective.

## 6. Conclusion

With the widespread use of network systems and the advancement of Internet technology, we enjoy the convenience brought by the Internet lifestyle, and at the same time, we also face many network security problems. According to the latest Internet security report, the security of today's network environment is getting worse and worse, and the threats are becoming more and more complex and diverse, affecting people's normal life. In addition to the traditional security protection technology, intrusion detection can also actively protect the system and play an important role in protecting information security. With the progress of artificial intelligence theory, the intrusion detection technology based on machine learning has also become a research hotspot. In recent years, the amount of network data has increased significantly, and network attacks have become more complex and diverse. Facing these new network security challenges, intrusion detection systems based on traditional machine learning algorithms (SVM, Bayesian, etc.) have problems such as unstable performance and long training time. At the same time, the research of deep learning technology has become a hot spot, and it is widely used in image classification, speech recognition, spam filtering, and other fields and has achieved remarkable results. At the same time, it also provides a new field and research methodology for intrusion analysis. It can be seen that the deep learning intrusion detection research model spans multiple disciplines and is a new direction of interdisciplinary research with very broad research potential. On the basis of the basic intrusion detection model research, a DBN-PBT-TSVM hybrid intrusion detection model based on DBN and TSVM is designed and proposed in detail, focusing on data preprocessing, exception processing, and other modules. Through a large number of comparison experiments with the traditional intrusion detection model, it proves that the model still has strong detection ability in the face of massive, high-dimensional, and nonlinear interference data, avoiding the slow detection speed and traditional low-level interference detection. The number of layers of deep neural networks and the number of nodes in each layer lack relevant theoretical guidance. Most of the cases are selected based on personal experience or a large number of experiments, so further research can be done on this. The research in this paper is mainly aimed at the experiments of KDD99 and NSL-KDD datasets, and there will definitely be more unpredictable problems in practical application that may affect the effect of this method. Therefore, it is necessary to verify and improve the problem in practical application scenarios.

## Figures and Tables

**Table 1:** Malware application permission and its description.

Malicious software	Important sensitive permissions	Describe	Index
ADED	INTERNET, ACCESS_NETWOEK_STATE, R	Worm	0.315
	ECEIVE_BOOT_COMPLETED		
DeoidDewam	CHANGE_WIFI_STATE	Root uses a Trojan horse to raise rights	1.213
Bgserv	INTERNET, ERCEIVE_SMS, SEND_SMS	Worm	0.315
DroidDreamLight	INTERNET, READ_PHONE_STATE	Information stealing Trojan	1.521
Genimi	INTERNET, SEND_SMS	Worm	0.315
Pjapps	INTERNET, RECEIVE_SMS	Worm	0.315
Zsone	RECEIVE_SMS, SEND_SMS	Send SMS maliciously	0.625

**Table 2:** Comparison of intrusion detection accuracy rates of different feature learning methods.

Data set	PCA	Gain ration	DBN
S1	82.64	82.14	91.68
S2	83.15	83.06	93.87
S3	83.49	82.65	94.94
S4	83.96	82.54	95.45

**Table 3:** Protocol type encoding method.

Character attributes	Coded value
TCP	3
UDP	2
ICMP	1

**Table 4:** Experimental graph of hidden nodes of autoencoder.

Hidden node	Error	Operation hours
5	0.0369	0.03
10	0.0633	0.17
15	0.0689	0.33
20	0.0553	0.46
25	0.0426	0.56
30	0.1055	0.72
35	0.1437	0.88

**Table 5:** Experimental graph of hidden nodes selected by autoencoder.

Hidden node	Error	Operation hours
5	0.0353	0.03
6	0.0278	0.04
7	0.0269	0.07
8	0.0285	0.11
9	0.0311	0.14

**Table 6:** Comparison of detection rate with other feature extraction algorithms.

Algorithm	Detection rate (%)
PCA algorithm	96.3
SAE algorithm	98.4
Information gain algorithm	97.8
C4.5 algorithm	95.7

**Table 7:** BP network detection rate comparison with other optimization methods [30].

Detection method	Connection record								
	Normal record	Attack record				Detection rate (%)			Detection time (s)
		Dos	Probe	U2R	R2L				
	835	500	200	15	100	Rc	Rw	Rl	
BP	789	462	169	8	76	91.15	5.51	12.23	19.84
POS-BP	817	491	178	10	87	95.94	2.23	6.01	21.09
GA-BP	823	487	185	11	89	96.67	1.47	5.33	20.18
DBN-BP	826	496	189	10	93	97.82	1.13	3.36	21.25

## Data Availability

The data that support the findings of this study are available from the corresponding author upon reasonable request.

## References

[1] Ogiela L., Ogiela M. R., Ko H. (2020). Intelligent data management and security in cloud computing. *Sensors*.

[2] Namasudra S., Roy P. (2018). PpBAC. *Journal of Organizational and End User Computing*.

[3] Elhoseny M., Ramirez-Gonzalez G., Abu-Elnasr O. M., Shawkat S. A., Arunkumar N., Farouk A. (2018). Secure medical data transmission model for IoT-based healthcare systems. *IEEE Access*.

[4] Abdolmaleky M., Naseri M., Batle J., Farouk A., Gong L.-H. (2017). Red-Green-Blue multi-channel quantum representation of digital images. *Optik*.

[5] Xia Z., Wang X., Zhang L., Sun X., Qin Z., Ren K. (2017). A privacy-preserving and copy-deterrence content-based image retrieval scheme in cloud computing. *IEEE Transactions on Information Forensics and Security*.

[6] Deng R., Lu R., Lai C., Luan H. T., Liang H. (2017). Optimal workload allocation in fog-cloud computing toward balanced delay and power consumption. *IEEE Internet of Things Journal*.

[7] Wei W., Fan X., Song H., Fan, Yang J. (2018). Imperfect information dynamic stackelberg game based resource allocation using hidden markov for cloud computing. *IEEE Transactions on Services Computing*.

[8] Hirai T., Masuyyama H., Kasahara S., Takahashi Y. (2017). Performance analysis of large-scale parallel-distributed processing with backup tasks for cloud computing. *Journal of Industrial and Management Optimization*.

[9] Chen Y., Lin Z., Xing Z., Wang G., Gu Y. (2017). Deep learning-based classification of hyperspectral data. *Ieee Journal of Selected Topics in Applied Earth Observations and Remote Sensing*.

[10] Kermany D. S., Goldbaum M., Cai W. (2018). Identifying medical diagnoses and treatable diseases by image-based deep learning. *Cell*.

[11] Wang Q. (2021). Tennis online teaching information platform based on android mobile intelligent terminal. *Mobile Information Systems*.

[12] Jcn A., Csy B., Jkh C., Shiu L. C. (2019). Combining non-invasive wearable device and intelligent terminal in HealthCare IoT. *Procedia Computer Science*.

[13] Gao F., Ji S., Guo J. (2021). ID-Net: an improved mask R-CNN model for intrusion detection under power grid surveillance. *Neural Computing & Applications*.

[14] Huang Y. A., Wu H., Liu H., Yin Z. Lecture Notes in Computer Science Intelligent Robotics and Applications Volume.

[15] Aleesa A. M., Zaidan B. B., Zaidan A. A., Sahar N. M. (2020). Review of intrusion detection systems based on deep learning techniques: coherent taxonomy, challenges, motivations, recommendations, substantial analysis and future directions. *Neural Computing & Applications*.

[16] El-Hasnony I. M. E. (2019). Intelligent differential evolution based feature selection with deep neural network for intrusion detection in wireless sensor networks. *Journal of Intelligent Systems and Internet of Things*.

[17] Li Y., Wang Y., Hao S. (2019). Intelligent terminal face spoofing detection algorithm based on deep belief network. *Journal of Electronic Imaging*.

[18] Oshea T., Hoydis J. (2017). An introduction to deep learning for the physical layer. *IEEE Transactions on Cognitive Communications & Networking*.

[19] Ravi D., Wong C., Deligianni F. (2017). Deep learning for health informatics. *IEEE Journal of Biomedical and Health Informatics*.

[20] Tom Y., Devamanyu H., Soujanya P. (2018). Recent trends in deep learning based natural language processing [review article]. *IEEE Computational Intelligence Magazine*.

[21] Naseri M., Raji M. A., Hantehzadeh M. R., Farouk A., Boochani A., Solaymani S. (2015). A scheme for secure quantum communication network with authentication using GHZ-like states and cluster states controlled teleportation. *Quantum Information Processing*.

[22] Ramezani Mayiami M., Hajimirsadeghi M., Skretting K., Dong X., Blum R. S., Poor H. V. (2021). Bayesian Topology Learning and noise removal from network data. *Discover Internet of Things*.

[23] Maseleno A. (2019). Design of optimal machine learning based cybersecurity intrusion detection systems. *Journal of Cybersecurity and Information Management*.

[24] Wang P., Kong X., Peng G., Li X., Wang Z. Network Intrusion Detection Based on Deep Learning.

[25] Zhu X. X., Tuia D., Mou L., Xia G. S., Zhang L., Xu F. (2018). Deep learning in remote sensing: a comprehensive review and list of resources. *IEEE Geoscience & Remote Sensing Magazine*.

[26] Abulkasim H., Farouk A., Hamad S., Mashatan A., Ghose S. (2019). Secure dynamic multiparty quantum private comparison. *Scientific Reports*.

[27] Gordon H., Lyp T., Kimbro C., Tehranipoor S. (2021). A novel IoT sensor authentication using HaLo extraction method and memory chip variability. *Discover Internet of Things*.

[28] Goh G. B., Hodas N. O., Vishnu A. (2017). Deep learning for computational chemistry. *Journal of Computational Chemistry*.

[29] Codella N., Nguyen Q. B., Pankanti S. (2017). Deep learning ensembles for melanoma recognition in dermoscopy images. *IBM Journal of Research and Development*.

[30] (2021).

[31] Dong Y., Li J. (2017). Recent progresses in deep learning based acoustic models. *IEEE/CAA Journal of Automatica Sinica*.

[32] Sun X., Wu P., Hoi S. C. H. (2018). Face detection using deep learning: an improved faster RCNN approach. *Neurocomputing*.

[33] Jian Y., Ni J., Yang Y. (2017). Deep learning hierarchical representations for image steganalysis. *IEEE Transactions on Information Forensics and Security*.

[34] Zheng Z., Chen W., Wu X., Chen P. C. Y., Liu J. (2017). LSTM network: a deep learning approach for short-term traffic forecast. *IET Intelligent Transport Systems*.

[35] Burlina P., Pacheco K. D., Joshi N., Freund D. E., Bressler N. M. (2017). Comparing humans and deep learning performance for grading AMD: a study in using universal deep features and transfer learning for automated AMD analysis. *Computers in Biology and Medicine*.

[36] He L., Ota K., Dong M. (2018). Learning IoT in edge: deep learning for the Internet of things with edge computing. *IEEE Network*.

[37] Fang L., Cunefare D., Wang C., Guymer R. H., Li S., Farsiu S. (2017). Automatic segmentation of nine retinal layer boundaries in OCT images of non-exudative AMD patients using deep learning and graph search. *Biomedical Optics Express*.

[38] Zhao X., Zhou J. (2021). Power information network intrusion detection based on deep learning and cloud computing. *Journal of Intelligent and Fuzzy Systems*.

[39] Wang J., Jia Li, Zhang C. (2021). Intrusion detection in power information network based on deep learning and cloud computing. *Academic Journal of Engineering and Technology Science*.

